# Optimisation of the T-square sampling method to estimate population sizes

**DOI:** 10.1186/1742-7622-4-7

**Published:** 2007-06-01

**Authors:** Kristof Bostoen, Zaid Chalabi, Rebecca F Grais

**Affiliations:** 1 Department of Infectious and Tropical Diseases, London School of Hygiene and Tropical Medicine, Keppel Street, London, WC1E 7HT, UK; 2 Department of Public Health and Policy, London School of Hygiene and Tropical Medicine, Keppel Street, London, WC1E 7HT, UK; 3 Epicentre, 8 rue Saint Sabin, 75011 Paris, France

## Abstract

Population size and density estimates are needed to plan resource requirements and plan health related interventions. Sampling frames are not always available necessitating surveys using non-standard household sampling methods. These surveys are time-consuming, difficult to validate, and their implementation could be optimised. Here, we discuss an example of an optimisation procedure for rapid population estimation using T-Square sampling which has been used recently to estimate population sizes in emergencies. A two-stage process was proposed to optimise the T-Square method wherein the first stage optimises the sample size and the second stage optimises the pathway connecting the sampling points. The proposed procedure yields an optimal solution if the distribution of households is described by a spatially homogeneous Poisson process and can be sub-optimal otherwise. This research provides the first step in exploring how optimisation techniques could be applied to survey designs thereby providing more timely and accurate information for planning interventions.

## Background

There is a constant need to estimate population size and density for the purposes of planning resource requirements or assessing health needs. For reasons relating to timeliness, cost or practicality, data are often obtained through surveys that aim to collect representative samples. Public health specialists rely traditionally on detailed sample frames to survey populations. There are however many situations (such as those relating to displaced populations in emergencies) in which detailed sample frames are either unavailable or unfeasible. Only a small number of sampling methods are suitable for such situations.

Ecological methods, which often do not require a detailed sample frame, can offer practical solutions to household sampling problems and are currently being explored. These methods include sequential sampling techniques to estimate prevalence or program coverage [1,2], capture-recapture techniques [3,4], adaptive sampling [5], T-Square sampling [6] and Catana's wandering quarter method [7] to estimate population size and density.

One of the problems in validating and verifying sampling methods used in situations devoid of sampling frames is the difficulty in analysing the properties of the sampling methods [8]. Traditional optimisation of sampling methods is done using computationally intensive re-sampling techniques such as Monte Carlo (MC) or Latin Hypercube Sampling (LHS) simulations, while experimenting with different permutations of the parameters of the sampling method on simulated or real population data. Further, from a theoretical perspective, there are infinitely many scenarios (covering a wide distribution of household and individual data) for which the sampling method requires validation and verification.

**Figure 1:**
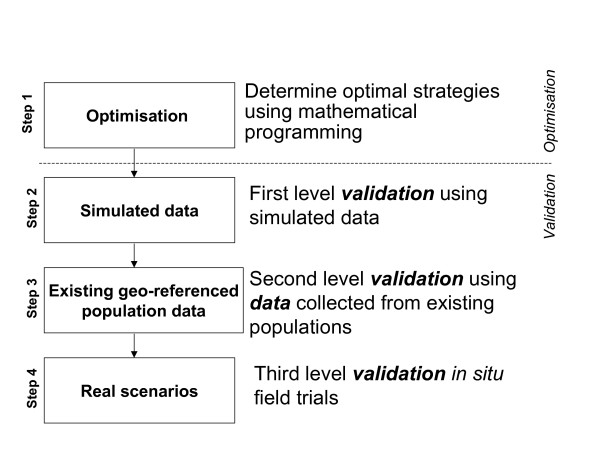
Validation steps of a household survey sampling method.

Mathematical Programming (MP) provides a powerful tool to optimise rigorously the properties of sampling methods [8]. The key advantage of MP is that it provides a more directed and less computing-intensive approach for optimisation compared to traditional methods. The purpose of this paper is to demonstrate this methodology in practice. Optimisation of a sampling method through MP could be considered as the first step in a four-step procedure for validation as shown in figure 1. Here, we explore optimisation as a first step in developing an alternative sampling method using the T-Square sampling method to estimate human population sizes as an example.

T-Square sampling is a distance-based sampling method whose statistical properties have been thoroughly investigated [9-14]. It has been used in ecology to estimate sizes, densities and deviations from random spatial distributions of mainly plant populations [15] and more recently it has been used to estimate the size of displaced human populations in emergency situations [6,16,17].

Estimating human populations in emergencies by using distance-based methods, such as the T-Square, rely on collecting data on distances between households (shelters) rather than on households *per se*. Advantages of distance sampling methods include:

• Human population density can be estimated even when not every household per unit area is detected;

• The same population density estimate can be calculated from data independently collected by multiple observers;

• A relatively small number of distances need to be measured;

• It may be less resource intensive and potentially more accurate than traditional sampling methods such as the quadrant method [6,16].

Two of the substantive issues to be addressed in this paper are whether:

• The assumptions on which the T-Square method is originally based for estimating plant population sizes are equally valid for estimating human population sizes;

• The T-Square method can be optimised.

## Analysis

### T-Square sampling and other distance-based methods

**Figure 2:**
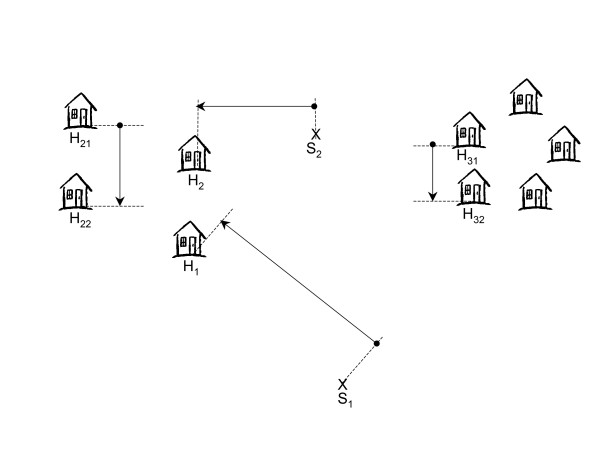
A schematic of distance-sampling methods. (Abbreviations: H, household; S, sampling points).

Two of the simplest distance-based methods to estimate population densities are those which measure distances between a random geographical point and its nearest household or a randomly selected household and its nearest neighbour. If the households are randomly distributed in the region of interest, both approaches are equivalent. On the other hand, if the households are aggregated, the assumption of randomness can be violated and both methods are prone to bias. However, the bias of the two methods in estimating population densities tends to be in opposite directions. This is because when households are aggregated, the average distance from a 'random geographic point to the nearest household' increases while the average distance measured between a 'random household to its nearest neighbour' decreases (figure 2). Using both distances together improves the robustness of the estimation method compared to the use of any estimation method which relies on either distance measure on its own.

**Figure 3:**
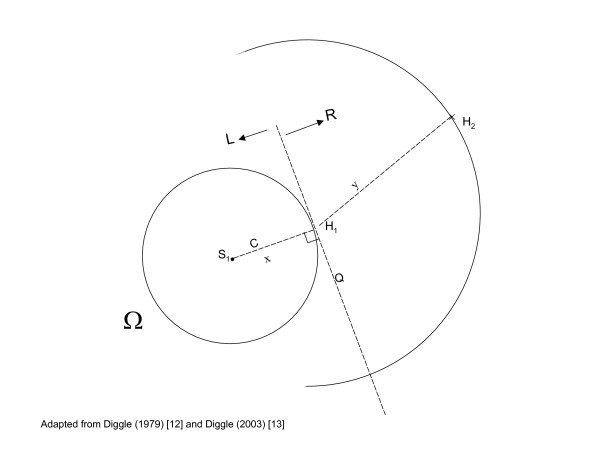
T-Square sampling method. (Abbreviations: H, household; S, sampling points; distances labelled *x *and *y*; planes labelled L and R; lines labelled Q and C; Ω, region of interest).

The T-Square method starts with generating random geographical points in the region of interest (Ω) such as point *S*_1 _in figure 3. From each point, the distance *x *is measured to the nearest household *H*_1 _along the line *C *connecting *S*_1 _and *H*_1_. At *H*_1 _the area is split, by a line *Q *which goes through *H*_1 _and is perpendicular to line *C*, in two planes *L *and *R*. The distance *y *from *H*_1 _to the nearest household in the opposite plane *R *(plane which does not contain point *S*_1_) is measured. The "T" formed by lines *C *and *Q *gives the method its name. The calculation of the population size and population densities based on these distances is explained in detail in Appendix I. The T-Square method assumes "complete spatial randomness". In mathematical terms, this assumption means that the households are described by a spatially homogeneous Poisson process (Appendix I).

**Figure 4:**
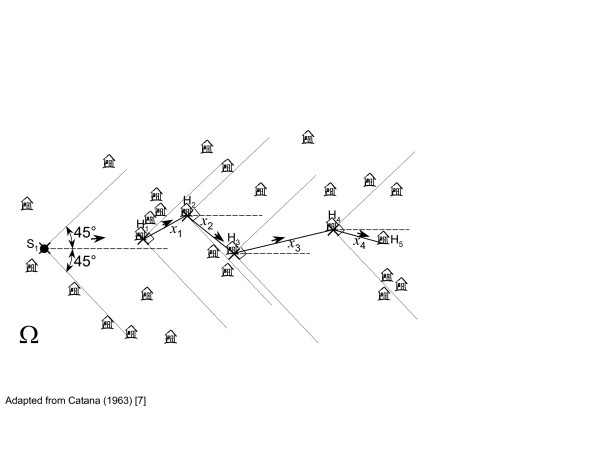
Catana's wandering quarter sampling method (Abbreviations: H, household; S, sampling points; *x*, distance).

An alternative method to T-Square sampling is Catana's 'wandering quarter' method [7]. The principle of the method is illustrated in figure 4. A transect of random direction and a random starting point (*S*_1_) is selected. From this point, the closest household (*H*_1_) within a 90° vertex (area bounded by the dotted lines) is determined. Starting from this household, the next household (*H*_2_) is selected in the same way resulting in a sequence of distances (*x*_1_, *x*_2_,...). This process is continued until the nearest household is outside the survey area. Although the properties of this method have not been thoroughly studied as those of T-Square sampling, Catana's method does not require the assumption of complete spatial randomness [7,13].

Choosing the appropriate distance-based method for use in human populations requires careful practical and theoretical considerations. Distances within which a surveyor can determine accurately the closest household from a random point or the closest household from a previously selected household are limited. In practice, it could be difficult to identify precisely the location of a household that occupies a large area. Furthermore some sampling methods are more sensitive than others to errors in the measurement of angles and distances. In the T-Square method the sample observations are pre-determined, unlike the wandering quarter method. The wandering quarter method could therefore be more difficult to plan in advance compared to the T-Square method if health data are to be collected from each household.

In addition to T-Square sampling and the Catana's wandering quarter methods, there are other distance-based methods such as the line-transect and point-transect distance methods [18,19]. It could be argued that although these methods are well established for estimating abundance of biological populations (plants or animals), extrapolating their use to household surveys would require evaluation. We note however that distance-based methods do not replace classical sampling methods where sample frames are available.

### Optimisation of the T-Square sampling method

The elements of optimising any household sampling method are the objective function (performance measure) to be optimised (maximised or minimised), the parameters of the method which can be tuned to optimise the objective function, and the constraints that are imposed on the values of these parameters [8]. In the context of optimising the T-Square method this is translated as follows.

The choice of the objective function to be optimised is not arbitrary and should be carefully considered. In real-life applications, a set of empirically-derived objective functions would be proposed and tailored to particular situations. Appendix II derives a simple objective function based on practical considerations. We present several examples of objective functions in the following paragraphs.

The simplest objective functions to be optimised (minimised in this case) are the standard error of the estimate of the average area per household (*E*) or the "cost" of the sampling (*C*), defined in a generic sense, as a measure of the "quantity of resources" required for sampling (for example, human resources). We can define an objective function which combines both those functions: *T *= *E *+ *αC *where *α *is a trade-off scalar, or parameter, which has a dual purpose: to scale *E *and *C *numerically to the same unit and to weight the relative significance of each of them in terms of the overall performance measure.

An obvious parameter to tune is the number of sampling points (*m*). Both terms (*E *and *C*) in the above combined objective function depend on *m*. We would expect *E*(*m*) to decrease monotonically with respect to *m *and *C*(*m*) to increase monotonically with *m *thus providing a trade-off in the choice of *m *to be optimised.

A key assumption in the optimisation analysis is that the distribution of the households can be described adequately by a two-dimensional spatially homogeneous Poisson process (Appendix I). In using the T-Square method, there is a potential bias in the estimate of the household density (mean number of households per unit area) if the Poisson assumption does not hold. The standard error term *E*(*m*) is proportional to m−1
 MathType@MTEF@5@5@+=feaafiart1ev1aaatCvAUfKttLearuWrP9MDH5MBPbIqV92AaeXatLxBI9gBaebbnrfifHhDYfgasaacH8akY=wiFfYdH8Gipec8Eeeu0xXdbba9frFj0=OqFfea0dXdd9vqai=hGuQ8kuc9pgc9s8qqaq=dirpe0xb9q8qiLsFr0=vr0=vr0dc8meaabaqaciaacaGaaeqabaqabeGadaaakeaadaGcaaqaaiabd2gaTnaaCaaaleqabaGaeyOeI0IaeGymaedaaaqabaaaaa@3029@ provided the sampling points are well spaced. The constant of proportionality however will depend on the underlying distribution and therefore would influence the optimal solution. Unlike the expression for *E*(*m*), the expression of *C*(*m*) is derived from practical considerations. The constraints on *m *are usually in the form of simple bounds on the sample size, i.e. greater than zero, but less than 60.

For illustrative purposes, we chose the following objective function to be minimised as a first example:

T(m)=m−1+α m2
 MathType@MTEF@5@5@+=feaafiart1ev1aaatCvAUfKttLearuWrP9MDH5MBPbIqV92AaeXatLxBI9gBaebbnrfifHhDYfgasaacH8akY=wiFfYdH8Gipec8Eeeu0xXdbba9frFj0=OqFfea0dXdd9vqai=hGuQ8kuc9pgc9s8qqaq=dirpe0xb9q8qiLsFr0=vr0=vr0dc8meaabaqaciaacaGaaeqabaqabeGadaaakeaacqWGubavcqGGOaakcqWGTbqBcqGGPaqkcqGH9aqpdaGcaaqaaiabd2gaTnaaCaaaleqabaGaeyOeI0IaeGymaedaaaqabaGccqGHRaWkiiGacqWFXoqycqqGGaaicqWGTbqBdaahaaWcbeqaaiabikdaYaaaaaa@3B50@

**Figure 5:**
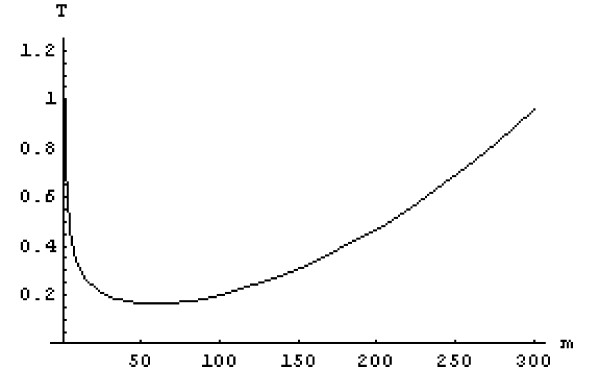
Objective function corresponding to Equation (1). (Abbreviations: T, objective function; *m*, number of sampling points).

The above objective function is the weighted sum of two terms: the standard error of the population size estimate and a quadratic cost relationship. The optimal sample size is sensitive to the choice of the trade-off parameter *α*. The choice of *α *balances the importance of maximising the precision of the estimate against minimising cost. In this example, we set *α *to 10^-5 ^and the simple bound constraint to 1 ≤ *m*. Figure 5 shows the variation of *T*(*m*) with *m*.

The minimisation was carried out in *Mathematica *using a standard non-linear programming optimisation algorithm [20]. The optimal sample size (to the nearest integer) is m* = 58.

Another example of an objective function was chosen to reflect a different cost-sample size relationship:

T(m)=m−1+α tanh⁡(βm)
 MathType@MTEF@5@5@+=feaafiart1ev1aaatCvAUfKttLearuWrP9MDH5MBPbIqV92AaeXatLxBI9gBaebbnrfifHhDYfgasaacH8akY=wiFfYdH8Gipec8Eeeu0xXdbba9frFj0=OqFfea0dXdd9vqai=hGuQ8kuc9pgc9s8qqaq=dirpe0xb9q8qiLsFr0=vr0=vr0dc8meaabaqaciaacaGaaeqabaqabeGadaaakeaacqWGubavcqGGOaakcqWGTbqBcqGGPaqkcqGH9aqpdaGcaaqaaiabd2gaTnaaCaaaleqabaGaeyOeI0IaeGymaedaaaqabaGccqGHRaWkiiGacqWFXoqycqqGGaaicyGG0baDcqGGHbqycqGGUbGBcqGGObaAcqGGOaakcqWFYoGycqWFGaaicqWGTbqBcqGGPaqkaaa@43BC@

**Figure 6:**
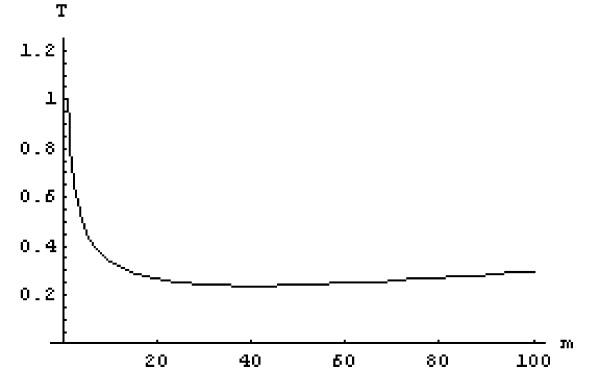
Objective function corresponding to Equation (2). (Abbreviations: T, objective function; *m*, number of sampling points).

The standard error term is the same as in the previous example, but the cost term is assumed to increase asymptotically with respect to sample size and is modelled using a hyperbolic tangent function where *β *is an empirically derived parameter. In the simulation, *β *is set to 0.002. This relationship represents scenarios where the incremental cost becomes smaller with progressively increasing sample size. The trade-off parameter *α *was set to unity and the same constraint was used as before. Figure 6 shows the variation of *T*(*m*) with *m*. The optimal sample size (to the nearest integer) in this example is m* = 40.

The two previous simulations were concerned with optimising sample size. Once the optimal sample size is determined, one can envisage a second optimisation stage whose aim is to select the optimal pathway for data collection. This could be required in practice for operational reasons and is not necessarily reflected in the cost function of the first stage optimisation problem. The optimal pathway is defined as the shortest pathway connecting all the sampling points. It is assumed here that one observer would be carrying out the survey.

**Figure 7:**
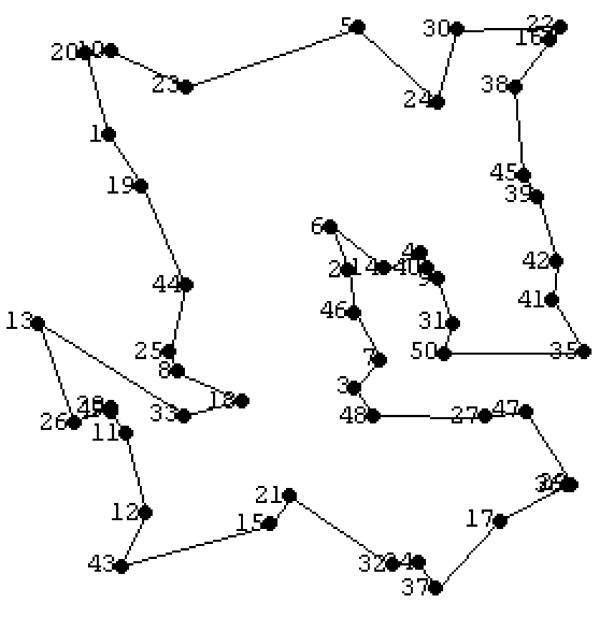
Location of sampling points.

Assume that the optimal sample size (obtained in the first optimisation step) is m* = 50. Figure 7 simulates a two-dimensional display of the 50 sampling points chosen randomly in a square plane whose boundary corner points have coordinates: (0,0), (0,5), (5,0) and (5,5). The two coordinates of each of the sampling points are generated independently using a pseudo random number generator. The random number generator produces a real number uniformly distributed between 0 and 5. Ignoring for the time being the straight-line segments, the dots numbered 1 to 50 in figure 7 represent the locations of the random points in the plane. Dot 1 is the location of the first sampling point selected, and dot 50 is the location of the last point selected.

The optimisation is concerned with computing the shortest pathway that connects all the sampling points. This is a very well known and classical problem in combinatorial optimization known as the "Travelling Salesperson Problem" [21]. The problem is to determine the least-distance route taken by a salesperson to visit a fixed number of cities in which each city is visited once only and in which the trip starts and ends at the same point. The Travelling Salesperson Problem (TSP) is not easy to solve (computational difficulty increases with the number of cities) and there is extensive literature on fast and efficient numerical algorithms used to solve both the classical version and more complex variations of the TSP [22,23].

Here, we solved the TSP problem in *Mathematica *[20,24]. The optimisation method used is called simulated annealing. Simulated annealing is a stochastic approach to find the global solution of an optimization problem where there could be multiple local solutions [25]. In this approach, an optimal solution is found iteratively by selecting randomly at each step a point in the neighbourhood of the current solution and then directing the search in the subsequent steps to improve the value of the objective function whilst not getting trapped in a local solution. It has been found that simulated annealing has several advantages over other optimization methods to solve TSP [26]. (Additional information and an illustration of simulated annealing [27]).

**Figure 8:**
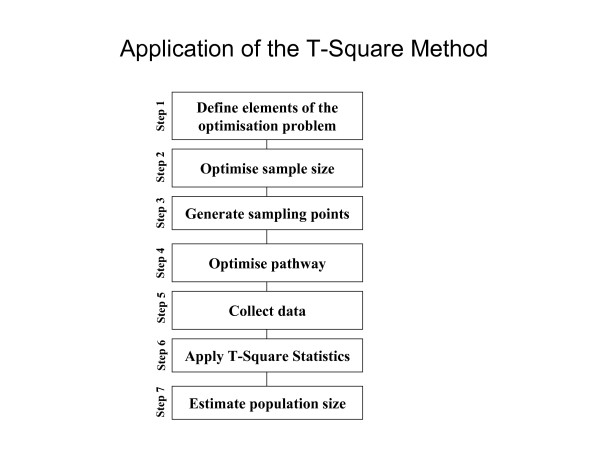
An illustration of the steps followed when applying the T-Square method in practice.

Figure 8 is a schematic diagram of a plausible sequence of steps to apply the optimised T-Square in practice. This is an extension of the chronology of steps proposed by Grais *et al *[6]. The first step defines the elements of the first optimisation problem, namely the standard error of the average area per household, the cost-sample size relationship and the constraints on the sample size. The second step solves for the optimal sample size. The third step generates the random coordinates of the sampling points bounded by the perimeter of domain Ω (the region of interest). The fourth step defines the optimal pathway. Starting from any sampling point on the optimal pathway and moving in either direction (clockwise or counter clockwise) the fifth step collates the pair of distances comprising: (i) The distance from the random sampling point to the nearest household and; (ii) The distance from that household to its nearest neighbour on the other side of the T-Square. The sixth step applies the T-Square statistics to test the null hypothesis that distribution of the households is completely random (Appendix I). If the null hypothesis is statistically not significant, the optimisation procedure yields a sub-optimal solution. Note that that the optimisation in *Step *2 is done only once whereas the optimisation is *Step *4 is required for each set of sampling points.

Because of the strict condition of complete randomness demanded by the T-Square sampling method, it is unlikely that this method would always be applicable. Catana's method could prove a valid alternative in the sense that it does not require complete spatial randomness however no results have been published for its use in human populations. As in the case of the T-Square method, Catana's method also has some restrictions in practice as discussed previously.

## Conclusion

The purpose of this paper was to illustrate the principle of optimising a household sampling method in situations where sampling frames are unavailable. We chose the T-Square method as the exemplar because it holds promise for estimating population sizes in such situations. The optimisation of the T-Square method was demonstrated using a simple illustrative example depicting scenarios that are faithful to the basic assumption of the method, namely that the distribution of the households can be described by a two-dimensional homogeneous Poisson process. If this assumption does not hold, then the proposed optimisation procedure would likely be sub-optimal. Further work should investigate optimising the T-Square method in scenarios that are more realistic and situations in which the distribution of the households is not described by a spatially inhomogeneous Poisson process.

The rigorous optimisation approach, which was demonstrated here on the T-Square method, can be applied to any other sampling method. Traditionally sampling methods were validated using computer simulations and were not formally optimised. The scope of the traditional computing-intensive approaches are somehow limited and the necessity of a mathematical approach for validation and optimisation is warranted [8].

Optimisation of sampling methods provides important information for surveys in contexts where sampling frames are not available. These techniques may be contained within computer software used by field survey teams without requiring technical knowledge of the algorithm. That is, a user-interface allowing survey teams to enter their objective function and generate an optimal survey strategy can mask formulae making them easier for use by non-technical survey teams. Instead of asking survey teams to define the objective function, they could be led through a set of heuristics which provide the number of points to be sampled. For example, in the case of the T-Square method, if the distribution of dwellings is uniform (e.g. as in a street-structured refugee camp) then sample *m*_1 _points, if the distribution of dwellings is clumped (e.g. as in a village-structured refugee camp) then sample *m*_2 _points. Another way to envision this step would be to ask a similar set of heuristics which are then translated into an objective function behind the user-interface. The second stage of optimisation, the travelling salesperson problem, could be contained within computer software and adapted for use in the field. These heuristics could be tailored to the key issues at hand in other sampling methods.

## Competing interests

The author(s) declare that they have no competing interests.

## Authors' contributions

KB and ZC conceived the study. All authors participated in drafting the manuscript. All authors read and approved the final manuscript.

## Appendix I. Statistical properties of the T-Square sampling method

The T-Square sampling method can be described simply in figure 3. We assume that individuals live in households that are not enumerated (i.e. there is no sampling frame). In emergencies, impromptu shelters grouped haphazardly represent households. Points *H*_1_, *H*_2 _and *H*_3 _represent the locations of three of the households. The region of interest (Ω) could contain *n *households (*H*_1_...*H*_*n*_). Point *S*_1 _represents an *arbitrary *chosen point in Ω. It represents one sample of *m *points (*S*_1_...*S*_*m*_), which are generated randomly and used as anchors for the estimation method.

Recall the description of figure 3. *C *is the straight line joining *S*_1 _to the nearest household (*H*_1_). *Q *is the line perpendicular to *C *at household *H*_1_. *Q *partitions the Ω plane into two semi-planes *R *and *L *indicated by the arrows. Household *H*_2 _is the nearest to *H*_1 _on the *R *semi-plane. The distance between *S*_1 _and *H*_1_, and the distance between *H*_1 _and *H*_2 _are denoted by *x *and *y*, respectively.

The primary assumption of the T-Square method is that the objects of interest (plants or households) are distributed randomly within the region of interest which means that their spatial distribution is described by a two-dimensional homogeneous Poisson point process [11,12]. This means that for any two non-overlapping regions A and B (within Ω) of areas *δ*_A _and *δ*_B _respectively, the probabilities of finding *k *households in A and B are statistically independent and that each probability is proportional to the area size:

p(NA=k)=exp⁡(−λδA)×(λδA)kk!p(NB=k)=exp⁡(−λδB)×(λδB)kk!
 MathType@MTEF@5@5@+=feaafiart1ev1aaatCvAUfKttLearuWrP9MDH5MBPbIqV92AaeXatLxBI9gBaebbnrfifHhDYfgasaacH8akY=wiFfYdH8Gipec8Eeeu0xXdbba9frFj0=OqFfea0dXdd9vqai=hGuQ8kuc9pgc9s8qqaq=dirpe0xb9q8qiLsFr0=vr0=vr0dc8meaabaqaciaacaGaaeqabaqabeGadaaakeaafaqabeGabaaabaGaemiCaa3aaeWaaeaacqWGobGtdaWgaaWcbaGaemyqaeeabeaakiabg2da9iabdUgaRbGaayjkaiaawMcaaiabg2da9maalaaabaGagiyzauMaeiiEaGNaeiiCaa3aaeWaaeaacqGHsisliiGacqWF7oaBcqWF0oazdaWgaaWcbaGaemyqaeeabeaaaOGaayjkaiaawMcaaiabgEna0oaabmaabaGae83UdWMae8hTdq2aaSbaaSqaaiabdgeabbqabaaakiaawIcacaGLPaaadaahaaWcbeqaaiabdUgaRbaaaOqaaiabdUgaRjabcgcaHaaaaeaacqWGWbaCdaqadaqaaiabd6eaonaaBaaaleaacqWGcbGqaeqaaOGaeyypa0Jaem4AaSgacaGLOaGaayzkaaGaeyypa0ZaaSaaaeaacyGGLbqzcqGG4baEcqGGWbaCdaqadaqaaiabgkHiTiab=T7aSjab=r7aKnaaBaaaleaacqWGcbGqaeqaaaGccaGLOaGaayzkaaGaey41aq7aaeWaaeaacqWF7oaBcqWF0oazdaWgaaWcbaGaemOqaieabeaaaOGaayjkaiaawMcaamaaCaaaleqabaGaem4AaSgaaaGcbaGaem4AaSMaeiyiaecaaaaaaaa@6CC3@

In Equation (I.1), *N*_A _and *N*_B _are respectively the number of households in regions A and B, and *λ *is the density (number of households per unit area) of the underpinning Poisson process and the parameter to be estimated.

Of course, the principal assumption of the T-Square method is very restrictive in the context of human population estimates. There are several statistical tests available to test for complete randomness of spatial point patterns [9,12-14,28-31]. The relaxation of this assumption has implications for the robustness of the method (see below) used to estimate *λ *[12].

Recall that *x *is the distance between point *S*_1 _and household *H*_1_. Consider next the ensemble of all such distances between the randomly chosen sample points (*S*_1_...*S*_*m*_) and their nearest households (*H*_1_...*H*_*m*_) and assume for simplicity that *n *= *m*. The probability density function (*pdf*) of *x *is [9,31]

*f*(*x*) = 2*π λ x *exp(-*π λ x*^2^)

It follows from Equation (I.2) that the random variable *ω *defined by *ω *= 2*π λ x*^2 ^is chi-square (*χ*^2^) distributed with 2 degrees of freedom [12].

If we selected the households arbitrarily, instead of the sampling points, and measured the distance between each selected household and its nearest neighbour, this distance will have the same *pdf *as *x*. However, households cannot be selected arbitrarily without enumeration of these households.

Distance methods invariably use pairs of distances between each of the random points and the nearest household and the distances between those households and their nearest neighbours (defined in some sense). With reference to figure 3, this means that the pair (*x*, *y*) could be used to estimate *λ*. Besag and Gleaves [9,12] showed that under the principal assumption that the households are distributed as a homogeneous Poisson process, y2
 MathType@MTEF@5@5@+=feaafiart1ev1aaatCvAUfKttLearuWrP9MDH5MBPbIqV92AaeXatLxBI9gBaebbnrfifHhDYfgasaacH8akY=wiFfYdH8Gipec8Eeeu0xXdbba9frFj0=OqFfea0dXdd9vqai=hGuQ8kuc9pgc9s8qqaq=dirpe0xb9q8qiLsFr0=vr0=vr0dc8meaabaqaciaacaGaaeqabaqabeGadaaakeaadaWcaaqaaiabdMha5bqaamaakaaabaGaeGOmaidaleqaaaaaaaa@2F44@ is independent of *x *and identically distributed to it. In other words, y2
 MathType@MTEF@5@5@+=feaafiart1ev1aaatCvAUfKttLearuWrP9MDH5MBPbIqV92AaeXatLxBI9gBaebbnrfifHhDYfgasaacH8akY=wiFfYdH8Gipec8Eeeu0xXdbba9frFj0=OqFfea0dXdd9vqai=hGuQ8kuc9pgc9s8qqaq=dirpe0xb9q8qiLsFr0=vr0=vr0dc8meaabaqaciaacaGaaeqabaqabeGadaaakeaadaWcaaqaaiabdMha5bqaamaakaaabaGaeGOmaidaleqaaaaaaaa@2F44@ has the same *pdf *as *x *(Equation I.2). Using this statistical feature of the distribution of the pair of variables (*x*, *y*), a robust estimator for *λ *is [12]

η=π×(∑i=1mxi2+12∑i=1myi2)2mλ=η−1
 MathType@MTEF@5@5@+=feaafiart1ev1aaatCvAUfKttLearuWrP9MDH5MBPbIqV92AaeXatLxBI9gBaebbnrfifHhDYfgasaacH8akY=wiFfYdH8Gipec8Eeeu0xXdbba9frFj0=OqFfea0dXdd9vqai=hGuQ8kuc9pgc9s8qqaq=dirpe0xb9q8qiLsFr0=vr0=vr0dc8meaabaqaciaacaGaaeqabaqabeGadaaakeaafaqaaeGabaaabaacciGae83TdGMaeyypa0Jae8hWdaNaey41aq7aaSaaaeaadaqadaqaamaaqahabaGaemiEaG3aa0baaSqaaiabdMgaPbqaaiabikdaYaaaaeaacqWGPbqAcqGH9aqpcqaIXaqmaeaacqWGTbqBa0GaeyyeIuoakiabgUcaRmaalaaabaGaeGymaedabaGaeGOmaidaamaaqahabaGaemyEaK3aa0baaSqaaiabdMgaPbqaaiabikdaYaaaaeaacqWGPbqAcqGH9aqpcqaIXaqmaeaacqWGTbqBa0GaeyyeIuoaaOGaayjkaiaawMcaaaqaaiabikdaYiabd2gaTbaaaeaacqWF7oaBcqGH9aqpcqWF3oaAdaahaaWcbeqaaiabgkHiTiabigdaXaaaaaaaaa@563A@

where *η *is the average area per household.

The principal assumption can be tested using appropriate T-Square sampling statistical tests [9,11,14]. These statistical tests are used to test the null hypothesis that the households (or shelters) are distributed as a homogeneous two-dimensional Poisson process. Under the null hypothesis the random variable on the left hand side of Equation (I.4) [6,9,11]

z=(t−12)(12m)−1
 MathType@MTEF@5@5@+=feaafiart1ev1aaatCvAUfKttLearuWrP9MDH5MBPbIqV92AaeXatLxBI9gBaebbnrfifHhDYfgasaacH8akY=wiFfYdH8Gipec8Eeeu0xXdbba9frFj0=OqFfea0dXdd9vqai=hGuQ8kuc9pgc9s8qqaq=dirpe0xb9q8qiLsFr0=vr0=vr0dc8meaabaqaciaacaGaaeqabaqabeGadaaakeaacqWG6bGEcqGH9aqpdaWcaaqaamaabmaabaGaemiDaqNaeyOeI0YaaSaaaeaacqaIXaqmaeaacqaIYaGmaaaacaGLOaGaayzkaaaabaWaaOaaaeaacqGGOaakcqaIXaqmcqaIYaGmcqWGTbqBcqGGPaqkdaahaaWcbeqaaiabgkHiTiabigdaXaaaaeqaaaaaaaa@3C29@

is normally distributed with zero mean and unit variance, where

t=∑i=1m(xi2xi2+12yi2)m
 MathType@MTEF@5@5@+=feaafiart1ev1aaatCvAUfKttLearuWrP9MDH5MBPbIqV92AaeXatLxBI9gBaebbnrfifHhDYfgasaacH8akY=wiFfYdH8Gipec8Eeeu0xXdbba9frFj0=OqFfea0dXdd9vqai=hGuQ8kuc9pgc9s8qqaq=dirpe0xb9q8qiLsFr0=vr0=vr0dc8meaabaqaciaacaGaaeqabaqabeGadaaakeaacqWG0baDcqGH9aqpdaWcaaqaamaaqahabaWaaeWaaeaadaWcaaqaaiabdIha4naaDaaaleaacqWGPbqAaeaacqaIYaGmaaaakeaacqWG4baEdaqhaaWcbaGaemyAaKgabaGaeGOmaidaaOGaey4kaSYaaSaaaeaacqaIXaqmaeaacqaIYaGmaaGaemyEaK3aa0baaSqaaiabdMgaPbqaaiabikdaYaaaaaaakiaawIcacaGLPaaaaSqaaiabdMgaPjabg2da9iabigdaXaqaaiabd2gaTbqdcqGHris5aaGcbaGaemyBa0gaaaaa@47FC@

As was argued by Diggle [12] and proposed in practice for use in human population estimates by Grais *et al *[6], hypothesis testing can be carried out as a two-step procedure. In the first step, the above null hypothesis is tested for statistical significance and if found to be statistically not significant, a supplementary null hypothesis is tested for statistical significance. In this second step, the null hypothesis corresponds to *u*^2 ^being *χ*^2^-distributed with *m *- 1 degrees of freedom where

u=48m13m+1×(mLog(∑i=1m(xi2+12yi2))−∑i=1mLog(xi2+12yi2))
 MathType@MTEF@5@5@+=feaafiart1ev1aaatCvAUfKttLearuWrP9MDH5MBPbIqV92AaeXatLxBI9gBaebbnrfifHhDYfgasaacH8akY=wiFfYdH8Gipec8Eeeu0xXdbba9frFj0=OqFfea0dXdd9vqai=hGuQ8kuc9pgc9s8qqaq=dirpe0xb9q8qiLsFr0=vr0=vr0dc8meaabaqaciaacaGaaeqabaqabeGadaaakeaacqWG1bqDcqGH9aqpdaWcaaqaaiabisda0iabiIda4iabd2gaTbqaaiabigdaXiabiodaZiabd2gaTjabgUcaRiabigdaXaaacqGHxdaTdaqadaqaaiabd2gaTjabdYeamjabd+gaVjabdEgaNnaabmaabaWaaabCaeaadaqadaqaaiabdIha4naaDaaaleaacqWGPbqAaeaacqaIYaGmaaGccqGHRaWkdaWcaaqaaiabigdaXaqaaiabikdaYaaacqWG5bqEdaqhaaWcbaGaemyAaKgabaGaeGOmaidaaaGccaGLOaGaayzkaaaaleaacqWGPbqAcqGH9aqpcqaIXaqmaeaacqWGTbqBa0GaeyyeIuoaaOGaayjkaiaawMcaaiabgkHiTmaaqahabaGaemitaWKaem4Ba8Maem4zaC2aaeWaaeaacqWG4baEdaqhaaWcbaGaemyAaKgabaGaeGOmaidaaOGaey4kaSYaaSaaaeaacqaIXaqmaeaacqaIYaGmaaGaemyEaK3aa0baaSqaaiabdMgaPbqaaiabikdaYaaaaOGaayjkaiaawMcaaaWcbaGaemyAaKMaeyypa0JaeGymaedabaGaemyBa0ganiabggHiLdaakiaawIcacaGLPaaaaaa@6D8E@

If both hypotheses are statistically not significant (when the spatial pattern is described by a two-dimensional homogeneous Poisson process), it is justified to use Equation (I.3) to estimate the average area per household (*η*). The 95% confidence interval for *η *is calculated by:

I=[η−1.96×η2m,η+1.96×η2m]
 MathType@MTEF@5@5@+=feaafiart1ev1aaatCvAUfKttLearuWrP9MDH5MBPbIqV92AaeXatLxBI9gBaebbnrfifHhDYfgasaacH8akY=wiFfYdH8Gipec8Eeeu0xXdbba9frFj0=OqFfea0dXdd9vqai=hGuQ8kuc9pgc9s8qqaq=dirpe0xb9q8qiLsFr0=vr0=vr0dc8meaabaqaciaacaGaaeqabaqabeGadaaakeaacqWGjbqscqGH9aqpdaWadaqaaGGaciab=D7aOjabgkHiTiabigdaXiabc6caUiabiMda5iabiAda2iabgEna0oaalaaabaGae83TdGgabaWaaOaaaeaacqaIYaGmcqWGTbqBaSqabaaaaOGaeiilaWIae83TdGMaey4kaSIaeGymaeJaeiOla4IaeGyoaKJaeGOnayJaey41aq7aaSaaaeaacqWF3oaAaeaadaGcaaqaaiabikdaYiabd2gaTbWcbeaaaaaakiaawUfacaGLDbaaaaa@4AF4@

The implication is that the underlying assumptions concerning the distributions of the households (or shelters) may be violated as indicated by the statistical tests performed after field data were collected. In this case, a more robust estimate of *η *is [12,13]

η=πm×(∑i=1mxi2×12∑i=1myi2)
 MathType@MTEF@5@5@+=feaafiart1ev1aaatCvAUfKttLearuWrP9MDH5MBPbIqV92AaeXatLxBI9gBaebbnrfifHhDYfgasaacH8akY=wiFfYdH8Gipec8Eeeu0xXdbba9frFj0=OqFfea0dXdd9vqai=hGuQ8kuc9pgc9s8qqaq=dirpe0xb9q8qiLsFr0=vr0=vr0dc8meaabaqaciaacaGaaeqabaqabeGadaaakeaaiiGacqWF3oaAcqGH9aqpdaWcaaqaaiab=b8aWbqaaiabd2gaTbaacqGHxdaTdaGcaaqaamaabmaabaWaaabCaeaacqWG4baEdaqhaaWcbaGaemyAaKgabaGaeGOmaidaaOGaey41aq7aaSaaaeaacqaIXaqmaeaacqaIYaGmaaWaaabCaeaacqWG5bqEdaqhaaWcbaGaemyAaKgabaGaeGOmaidaaaqaaiabdMgaPjabg2da9iabigdaXaqaaiabd2gaTbqdcqGHris5aaWcbaGaemyAaKMaeyypa0JaeGymaedabaGaemyBa0ganiabggHiLdaakiaawIcacaGLPaaaaSqabaaaaa@5031@

Equation (I.3) (or Equation (I.8)) estimates the average area per household. The human population *ρ *in the region of interest (Ω) can be estimated by Equation (I.9) [6]

ρ=κ×Γη
 MathType@MTEF@5@5@+=feaafiart1ev1aaatCvAUfKttLearuWrP9MDH5MBPbIqV92AaeXatLxBI9gBaebbnrfifHhDYfgasaacH8akY=wiFfYdH8Gipec8Eeeu0xXdbba9frFj0=OqFfea0dXdd9vqai=hGuQ8kuc9pgc9s8qqaq=dirpe0xb9q8qiLsFr0=vr0=vr0dc8meaabaqaciaacaGaaeqabaqabeGadaaakeaaiiGacqWFbpGCcqGH9aqpcqWF6oWAcqGHxdaTdaWcaaqaaiabfo5ahbqaaiab=D7aObaaaaa@365C@

where *κ *is the average household population and Γ is total the area of region Ω.

## Appendix II. Objective function

This section describes a simple objective function which has been used in practice to determine sample size requirements in cluster surveys on provision of water, sanitation and hygiene. The cluster surveys used a two stage sampling approach. In the first stage the primary sampling units (PSUs) were selected with a probability proportioned to their size. In the second stage a simple random sample of size *b *was taken from each PSU, where *b *is the number of basic sampling units (BSUs) within each PSU. *b *is also known as the 'take'.

The objective function describes the relationship between the survey cost and number of BSUs. The total sample size (*s*) is determined by the number of clusters (*c*) and the number of BSUs (*s *= *c *× *b*). The cost of the total survey (*C*_*survey*_) is the sum of a fixed cost (*C*_*fixed*_) independent of *b *and a variable cost (*C*_*variable*_) which depends on *b *and *c*.

*C*_*survey *_= *C*_*fixed *_+ *C*_*variable*_

The variable cost is given

*C*_*variable *_= *c *× *C*_*PSU *_+ *c *× *b *× *C*_*BSU*_

where *C*_*PSU *_and *C*_*BSU *_are respectively the survey cost per PSU and per BSU. If we set Cratio=CPSUCBSU
 MathType@MTEF@5@5@+=feaafiart1ev1aaatCvAUfKttLearuWrP9MDH5MBPbIqV92AaeXatLxBI9gBaebbnrfifHhDYfgasaacH8akY=wiFfYdH8Gipec8Eeeu0xXdbba9frFj0=OqFfea0dXdd9vqai=hGuQ8kuc9pgc9s8qqaq=dirpe0xb9q8qiLsFr0=vr0=vr0dc8meaabaqaciaacaGaaeqabaqabeGadaaakeaacqWGdbWqdaWgaaWcbaGaemOCaiNaemyyaeMaemiDaqNaemyAaKMaem4Ba8gabeaakiabg2da9maalaaabaGaem4qam0aaSbaaSqaaiabdcfaqjabdofatjabdwfavbqabaaakeaacqWGdbWqdaWgaaWcbaGaemOqaiKaem4uamLaemyvaufabeaaaaaaaa@3F6C@ and assume without loss of generality that *C*_*BSU *_= 1 (i.e. represent all costs relative to *C*_*BSU*_), Equation (II.2) becomes

*C*_*variale *_= (*C*_*ratio *_+ *b*) × *c*

The required size of the cluster can be expressed in terms of the expected proportion of the target population, *p*, and the standard error of its mean estimate, *ξ *[32]

c=p×(1−p)ξ2×b×deff
 MathType@MTEF@5@5@+=feaafiart1ev1aaatCvAUfKttLearuWrP9MDH5MBPbIqV92AaeXatLxBI9gBaebbnrfifHhDYfgasaacH8akY=wiFfYdH8Gipec8Eeeu0xXdbba9frFj0=OqFfea0dXdd9vqai=hGuQ8kuc9pgc9s8qqaq=dirpe0xb9q8qiLsFr0=vr0=vr0dc8meaabaqaciaacaGaaeqabaqabeGadaaakeaacqWGJbWycqGH9aqpdaWcaaqaaiabdchaWjabgEna0kabcIcaOiabigdaXiabgkHiTiabdchaWjabcMcaPaqaaGGaciab=57a4naaCaaaleqabaGaeGOmaidaaOGaey41aqRaemOyaigaaiabgEna0kabdsgaKnaaBaaaleaacqWGLbqzcqWGMbGzcqWGMbGzaeqaaaaa@4571@

where *d*_*eff *_is the design effect [33]

*d*_*eff *_= 1 + *ρ *× (*b *- 1)

*ρ *is the rate of homogeneity. Substituting Equations (II.4) and (II.5) in (II.3) gives the expression of *C*_var*iable *_in terms of *b*

Cvar⁡iable=(Cratio+b)×p×(1−p)×(1+ρ×(b−1))ξ2×b
 MathType@MTEF@5@5@+=feaafiart1ev1aaatCvAUfKttLearuWrP9MDH5MBPbIqV92AaeXatLxBI9gBaebbnrfifHhDYfgasaacH8akY=wiFfYdH8Gipec8Eeeu0xXdbba9frFj0=OqFfea0dXdd9vqai=hGuQ8kuc9pgc9s8qqaq=dirpe0xb9q8qiLsFr0=vr0=vr0dc8meaabaqaciaacaGaaeqabaqabeGadaaakeaacqWGdbWqdaWgaaWcbaGagiODayNaeiyyaeMaeiOCaiNaemyAaKMaemyyaeMaemOyaiMaemiBaWMaemyzaugabeaakiabg2da9iabcIcaOiabdoeadnaaBaaaleaacqWGYbGCcqWGHbqycqWG0baDcqWGPbqAcqWGVbWBaeqaaOGaey4kaSIaemOyaiMaeiykaKIaey41aq7aaSaaaeaacqWGWbaCcqGHxdaTcqGGOaakcqaIXaqmcqGHsislcqWGWbaCcqGGPaqkcqGHxdaTcqGGOaakcqaIXaqmcqGHRaWkiiGacqWFbpGCcqGHxdaTcqGGOaakcqWGIbGycqGHsislcqaIXaqmcqGGPaqkcqGGPaqkaeaacqWF+oaEdaahaaWcbeqaaiabikdaYaaakiabgEna0kabdkgaIbaaaaa@651A@

**Figure 9:**
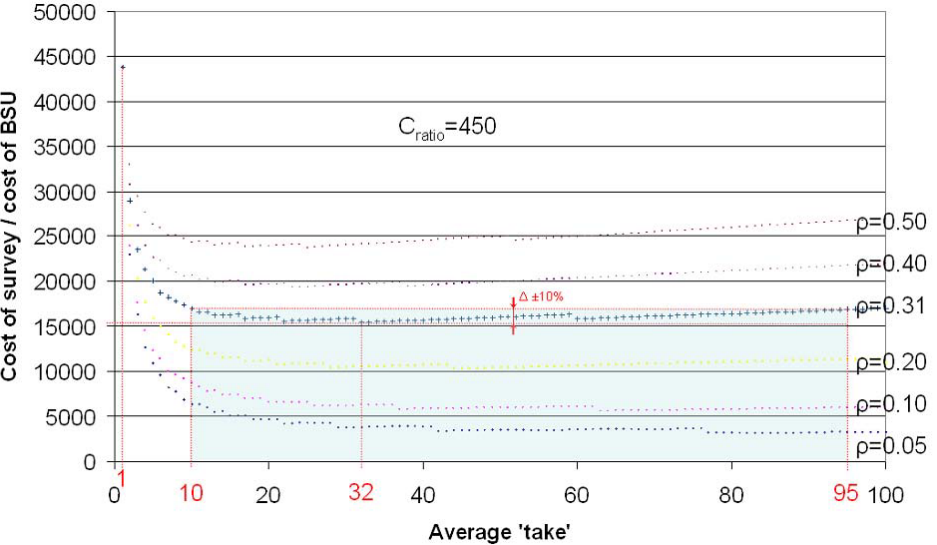
An example of a practically constructed objective function.

Figure 9 shows CsurveyCBSU
 MathType@MTEF@5@5@+=feaafiart1ev1aaatCvAUfKttLearuWrP9MDH5MBPbIqV92AaeXatLxBI9gBaebbnrfifHhDYfgasaacH8akY=wiFfYdH8Gipec8Eeeu0xXdbba9frFj0=OqFfea0dXdd9vqai=hGuQ8kuc9pgc9s8qqaq=dirpe0xb9q8qiLsFr0=vr0=vr0dc8meaabaqaciaacaGaaeqabaqabeGadaaakeaadaWcaaqaaiabdoeadnaaBaaaleaacqWGZbWCcqWG1bqDcqWGYbGCcqWG2bGDcqWGLbqzcqWG5bqEaeqaaaGcbaGaem4qam0aaSbaaSqaaiabdkeacjabdofatjabdwfavbqabaaaaaaa@3B3D@ in terms of *b*.

## References

[B1] Myatt M, Feleke T, Sadler K, Collins S (2005). A field trial of a survey method for estimating the coverage of selective feeding programmes. Bull World Health Organ.

[B2] Brooker S, Kabatereine NB, Myatt M, Russell Sothard J, Fenwick A (2005). Rapid assessment of schistosoma mansoni: the validity, applicability and cost-effectiveness of the Lot Quality Assurance Sampling Method in Uganda. Trop Med Int Health.

[B3] Luan R, Zeng G, Zhang D, Lou L, Yuan P, Liang P, Li Y (2005). A study on methods of estimating the population size of men who have sex with men in Southwest China. European Journal of Epidemiology.

[B4] Chao A, Tsay PK, Lin SH, Shau WY, Chao DY (2001). The applications of capture-recapture models to epidemiological data. Statist Med.

[B5] Martsolf DS, Courey TJ, Chapman TR, Draucker CB, Mims BL (2006). Adaptive sampling: recruting a diverse community sample of survivors of sexual violence. J Community Health Nurs.

[B6] Grais RF, Coulombier D, Ampuero J, Lucas MES, Barretto AT, Jacquier G, Diaz F, Balandine S, Mahoudeau C, Brown V (2006). Are rapid population estimates accurate? A field trial of two different assessment methods. Disasters.

[B7] Catana AJ (1963). The wandering quarter method of estimating population density. Ecology.

[B8] Bostoen K, Chalabi Z (2006). Optimising household survey sampling without sample frames. International Journal of Epidemiology.

[B9] Besag J, Gleaves JT (1973). On the detection of spatial pattern in plant communities. Bulletin of the International Statistical Institute.

[B10] Diggle PJ (1975). Robust density estimation using distance methods. Biometrika.

[B11] Diggle PJ (1977). The detection of random heterogeneity in plant populations. Biometrics.

[B12] Diggle PJ, Cormack RM, Ord JK (1979). Statistical methods for spatial point patterns in ecology. Spatial and temporal analysis in ecology.

[B13] Diggle PJ (2003). Statistical analysis of spatial point processes.

[B14] Diggle PJ, Besag J, Gleaves JT (1976). Statistical analysis of spatial point patterns by means of distance methods. Biometrics.

[B15] Young LJ, Young H (1998). Statistical ecology: a population perspective.

[B16] Brown V, Jacquier G, Coulombier D, Balandine S, Belanger F, Legros D (2001). Rapid assessment of population size by area sampling in disaster situations. Disasters.

[B17] Noji EK (2005). Estimating population size in emergencies. Bulletin of the World Health Organization.

[B18] Buckland ST, Anderson DR, Burnham KP, Laake JL (1993). Distance sampling: estimating abundance of biological populations.

[B19] Buckland ST, Anderson DR, Burnham KP, Laake JL, Borchers DL, Thomas L (2004). Advanced distance sampling. Estimating abundance of biological populations..

[B20] Wolfram S (2003). Mathematica, Fifth Edition.

[B21] Lawler EL, Lenstra JK, Rinnooy Kan AHG, Shmoys DB (1985). The traveling salesman problem. A guided tour of combinatorial optimization.

[B22] Moon C, Kim J, Choi G, Seo Y (2002). An efficient genetic algorithm for the traveling salesman problem with precedence constraints. European Journal of Operational Research.

[B23] Snyder LV, Daskin MS (2006). A random-key genetic algorithm for the genralized traveling salesman problem. European Journal of Operational Research.

[B24] Kripfganz J, Perlt H (2005). Operations Research 3.1. A Mathematica application package.

[B25] Pham DT, Karaboga D (2000). Intelligent optimization techniques. Genetic algorithms, Tabu search, simulated annealing and neural networks..

[B26] Nemhauser GL, Wolsey LA (1999). Integer and combinatorial optimization.

[B27] Simulated Annealing. http://www.cs.sandia.gov/opt/survey/sa.html.

[B28] Byth K, Ripley BD (1980). On sampling spatial patterns by distance methods. Biometrics.

[B29] Cormack RM (1977). The invariance of Cox and Lewis's statistic for the analysis of spatial patterns. Biometrika.

[B30] Hines WGS, O'Hara Hines RJ (1979). The Eberhardt statistic and the detection of nonrandomness of spatial point distributions. Biometrika.

[B31] Holgate P (1965). Tests of randomness based on distance methods. Biometrika.

[B32] Bennett S, Radalowicz A, Vella A, Tomkins A (1994). A computer simulation of household sampling schemes for health surveys in developing countries. International Journal of Epidemiology.

[B33] Kish L (1965). Survey sampling.

